# Research on influencing factors and improvement paths of rational medication literacy among college students: a cross-sectional survey in Zhejiang province

**DOI:** 10.3389/fpubh.2026.1775243

**Published:** 2026-04-10

**Authors:** Peiwen Wu, Han Fu, Biao Zhao, Mengting Zhang, Ziyu Chen, Dong He, Shucong Liu

**Affiliations:** School of Public Administration, Hangzhou Normal University, Hangzhou, China

**Keywords:** associative paths, college students, health education, influencing factors, rational medication literacy

## Abstract

**Introduction:**

Rational medication use is a critical component of public health and healthcare quality. However, college students, as a key transitional population in health behavior development, exhibit varying levels of medication literacy that remain underexplored. This study aims to investigate the level of rational medication literacy and health education acceptance among college students in Zhejiang Province, analyze the influencing factors and associative paths of medication literacy, and formulate targeted strategies to enhance rational medication literacy.

**Methods:**

A stratified convenience sampling method was used to select six representative universities in Zhejiang Province. A total of 1,500 students were surveyed, and 1,436 valid questionnaires were collected (95.73%). Non-parametric tests were employed for descriptive statistics. Univariate analysis and binary logistic regression identified influencing factors. Structural equation modeling was applied to explore the pathways between these factors and medication literacy.

**Results:**

The mean medication literacy score of the surveyed college students was 11.32 ± 4.75, with 796 participants (55.43%) achieving high-level literacy (score ≥ 13). Among the four dimensions of medication literacy, the non-prescription drug dimension received the highest mean score (3.65 ± 1.73); the drug advertisement dimension had the lowest (1.63 ± 1.04). The total effect sizes of variables on medication literacy were ranked by absolute value from largest to smallest as follows: long-term medication use (0.162), gender (0.160), social support (0.158), mother's educational level (−0.104), university attended (0.100), course selection (−0.095), academic workload (0.081), part-time work experience (0.073), monthly living expenses (0.026), major type (0.012). All six surveyed universities offered rational medication courses, but overall enrollment rates remained low. College students primarily obtained medical knowledge through internet media platforms (56.69%), followed by medical staff at campus clinics (48.96%).

**Discussion:**

The level of rational medication literacy among college students in Zhejiang Province requires further improvement. A multi-level health education network for rational medication use should be established, integrating self-directed learning, institutional promotion, and government guidance, with the goal of institutionalizing the cultivation of safe and rational medication awareness and enhancing students' health literacy, particularly in the domain of medication literacy.

## 1 Introduction

The rational use of medicine is the core component of China's pharmaceutical policy and a crucial element in the country's ongoing healthcare reform. The World Health Organization defines rational medicine use as “medicines that are appropriate for the patient's clinical needs, dosed according to the patient's individual requirements, administered for the appropriate duration, and at the lowest possible cost to the patient and their community.” This represents the ideal model for medicine therapy (1). However, in actual practice, irrational medication use frequently occurs. The WHO points out that one-third of patients worldwide die as a result of irrational medication use (2). Reports indicate that 12% to 32% of patients in China experience inappropriate medication use, with adverse drug reactions causing over 500,000 deaths annually (3).

Irrational medication use has become a major challenge in the global public health field. Antibiotic abuse, excessive infusion, blind self-medication and other phenomena will not only accelerate the spread of drug resistance, but also cause a large waste of medical resources and bring heavy economic burden. In addition, improper medication can directly affect the patient's treatment effect and medication safety, which may lead to serious adverse drug reactions, drug-induced diseases, and even lead to patient death. For patients with chronic diseases, long-term irrational medication will delay disease control, increase the risk of complications, prolong hospital stay, seriously affect their quality of life and long-term prognosis, and greatly reduce patients' trust in the medical and health system.

In 2022, China's National Health Commission issued the “Notice on Further Strengthening Drug Safety Management and Improving the Level of Rational Drug Use,” emphasizing the need to “further strengthen medicine safety management, enhance the level of rational medicine use, and safeguard medical quality and safety as well as the health rights and interests of the people (4). The impact of rational medicine use on public health has also prompted public reflection. By 2018, Pouliot et al. (5) had proposed a more widely accepted definition using the Delphi method: “Medication literacy refers to the extent to which individuals can access, understand, communicate, calculate, and process patient-specific information about their medications to make informed medication and health decisions, enabling safe and effective use of their medications regardless of the format in which the information is delivered (e.g., written, verbal, and visual).”

Improving the public's literacy in rational medication use is an important measure to promote the development of public health and ensure the safety of medication use, and it has significant practical significance. However, ordinary population groups exhibit considerable heterogeneity in terms of age range, cultural background, health status, occupation type, and medication needs, resulting in substantial differences within the group. This not only increases the difficulty of identifying factors that influence rational medication use literacy, but also makes it challenging to focus research results on modifiable factors, which is not conducive to accurately identifying core influencing factors and formulating targeted intervention strategies. In contrast, college students, as a high-quality young population group, have greater homogeneity in terms of age, educational background, and living environment, which can effectively reduce the interference of confounding factors on research results and improve the accuracy and reliability of the study. At the same time, college students are at a critical stage of establishing independent health behaviors and cognitive patterns, and their cognition and behaviors related to rational medication use are highly malleable. Conducting research on rational medication use literacy among this group can provide scientific evidence for universities to carry out health education and develop targeted intervention measures, while also guiding college students to establish scientific medication concepts and cultivate good medication behaviors, thereby fully leveraging their influence to indirectly improve the overall medication literacy of families and society.

Research has revealed that in recent years, Chinese college students have exhibited widespread irrational medication practices, including self-medication based on experience, repeated medication use, uncorrected medication use, failure to complete prescribed treatment courses, and arbitrary adjustment of dosage. Overall, their medication literacy remains relatively low (6–9). Therefore, comprehensively assessing college students' medication literacy is of paramount importance.

Zhejiang Province is located in the developed eastern region of China, boasting comprehensive academic disciplines. Its college student population is characterized by diverse regional origins and multifaceted lifestyles. Assessing the medication literacy of college students in Zhejiang Province and analyzing its influencing factors and pathways can help identify the characteristics and shortcomings of medication literacy among key populations in economically developed regions. This will facilitate the development of replicable intervention models, providing a reference for similar regions nationwide and advancing China's research on college students' medication literacy from regional surveys to targeted guidance.

This study was based on Bandura's theory of social learning, which posits that the formation of individual cognition and behavior is not the result of a single factor, but the product of the dynamic interaction among individual factors, environmental factors, and behavioral factors (10). Individuals' cognitive and behavioral abilities are gradually constructed and improved through continuous observation, imitation, reinforcement, and self-regulation in the social environment. Based on this theoretical framework, this study systematically summarized the various factors affecting college students' rational medication literacy into three categories according to the formation characteristics of college students' rational medication literacy, to ensure that the screening and classification of variables had clear theoretical support: First, personal factors, including gender, major type, maternal education level, monthly living expenses, etc.; these factors mainly reflect the individual's health cognition foundation and internal characteristics, and are the internal prerequisites that affect the formation of their medication literacy. The second is environmental factors, covering long-term medication use of family members, employment of family members in the pharmaceutical industry, level of social support, etc., which constitute the external reality for individuals to contact drug knowledge and learn rational drug use behavior, and are an important carrier for individuals to carry out observation, learning, and behavior reinforcement. The third is behavioral experience factors, including whether to take rational drug use courses, part-time work experience, academic workload perception, etc., which represent the individual's direct participation experience and practical experience in drug-related fields, and are the key path for their medication literacy to be consolidated and improved through practice.

From the perspective of social learning theory, social support, as a key environmental resource, can not only directly affect individuals' cognitive processes, influencing their reception and understanding of knowledge about rational medication use, but may also act as a mediating variable, transmitting the indirect effects of personal factors, environmental factors, and behavioral experience factors on college students' literacy in rational medication use. Based on this, the core research hypothesis of this study was proposed: the level of college students' literacy in rational medication use is not only directly influenced by personal factors, environmental factors, and behavioral experience factors, but may also be indirectly affected by these three types of factors through social support as a mediating variable.

To scientifically verify the aforementioned research hypotheses, this study intended to use structural equation modeling as the core analytical method. This method has unique methodological advantages, enabling the simultaneous handling of complex relationships among multiple independent variables, mediating variables, and dependent variables, accurately estimating the direct, indirect, and total effects between variables; at the same time, it tests the fit between the theoretical model and actual survey data through a multidimensional set of model fit indicators. Compared with traditional regression analysis, structural equation modeling can more deeply explore the internal mechanisms between variables, making it particularly suitable for examining the multi-factorial interactive pathways of the three major factors of individuals, environment, and behavior proposed by social learning theory, providing scientific and rigorous methodological support for the verification of theoretical hypotheses.

## 2 Materials and methods

### 2.1 Study design and sample

This study employed a stratified convenience sampling method during the survey period from December 2024 to February 2025. This study strictly stratified universities according to their disciplinary types. Based on the disciplinary layout and program characteristics of universities in Zhejiang Province, universities were divided into six categories: comprehensive, medical, science and engineering, normal (teacher-training), economics and management, and arts. One representative public undergraduate university was selected from each category for the survey, namely Zhejiang University, Zhejiang Chinese Medical University, Hangzhou Dianzi University, Hangzhou Normal University, Zhejiang Gongshang University, and Communication University of Zhejiang. During the student recruitment phase within each university, a cluster convenience sampling method was used. In collaboration with university and departmental faculty, online questionnaire QR codes were distributed to undergraduate classes of different years and majors. Surveyors who had received standardized training guided students on-site in completing the questionnaires and immediately verified the submission status to ensure the standardization of the survey process. The sampling framework of this study covered all full-time undergraduate students in the six universities mentioned above. The sample size was initially estimated at 860 using Kendall's sample size estimation method (10–20 times the number of questionnaire items). To avoid bias caused by invalid questionnaires, the sample size was further increased by 10%, ultimately leading to the distribution of 1,500 questionnaires. The study subjects were undergraduate students enrolled at six universities in Zhejiang Province. Inclusion criteria were: ① currently enrolled undergraduate students; ② individuals capable of understanding the questionnaire and willing to participate.

### 2.2 Ethics statement

This research protocol was approved by the Ethics Review Committee of Hangzhou Normal University (20240013) and conducted in accordance with the Declaration of Helsinki and ethical guidelines. All participants signed written informed consent forms to ensure the privacy and confidentiality of their personal information.

### 2.3 Measurements

The survey questionnaire consisted of two sections: basic information and the Medication Literacy Scale.

#### 2.3.1 Basic information

The items included gender, grade attended, major type, home address, monthly household income per capita, monthly living expenses, parents' educational level, employment status of household members, long-term medication use among household members, elective course selection, academic workload, part-time work experience, social support, and health education on rational medication use. Social support was assessed using the Social Support Rating Scale developed by Xiao Shuiyuan (11). The scale consists of 10 items covering three dimensions: (1) Objective Support (3 items), which mainly measures the material assistance and social network resources actually received by individuals in the past year, such as “In the past year, when you encountered emergencies or difficulties, what sources of financial support and practical help were available to you?”; (2) Subjective Support (4 items), which mainly evaluates the emotional experiences of being respected, understood, and supported as perceived by the individual, such as “How many close friends do you have who can provide support and help?”; (3) Utilization of Social Support (3 items), which examines the extent to which individuals actively seek and utilize social support when facing difficulties, such as “How do you seek help when you encounter troubles?”. The total score of the scale is 66 points. A total score above 44 is considered high-level social support, a total score between 22 and 44 is considered medium-level social support, and a total score of 22 or below is considered low-level social support. Higher scores indicate higher levels of social support.

#### 2.3.2 Medication literacy

The primary instrument used was the Chinese Rational Medication Literacy Assessment Scale (12), developed by the Yeh team at the College of Pharmacy, Taipei Medical University, specifically for the adult population in Taiwan. This scale comprised 17 items across four dimensions: vocabulary (5 items), non-prescription drugs (5 items), prescription drugs (4 items), and drug advertisements (3 items). It employed a dichotomous scoring method—correct answers received 1 point and incorrect answers 0 points—with a maximum score of 17. A total score of 13 or higher indicated a high level of medication literacy, whereas a score below 13 indicated a low level (13). In the original study, the scale's Cronbach's alpha coefficient was 0.72. This study assessed the reliability of the scale using Cronbach's alpha coefficient, yielding an overall alpha coefficient of 0.895. Exploratory factor analysis was employed to evaluate validity, with KMO values of 0.894 and *p* < 0.01, indicating that the scale possessed good reliability and validity.

### 2.4 Data collection

The distribution and collection of the questionnaires for this study were carried out by interviewers who had received standardized training. This survey was conducted through the online questionnaire platform “Wenjuanxing,” where respondents scanned a QR code to complete the questionnaire online; after completion, the interviewers verified the submission status on the spot and conducted a preliminary check of the completeness of the responses. After the questionnaires were collected, the researchers imported the data into Excel for organization and excluded invalid samples based on the following criteria: ① completion time of less than 30 s; ② repeated submissions from the same IP address; ③ responses containing obvious logical contradictions. These quality control measures effectively ensured the reliability and validity of the research data. In addition, small gifts were given to respondents during the survey to enhance their cooperation and ensure the smooth implementation of the questionnaire distribution and collection. A total of 1,512 students were invited to participate in this study, of whom 12 explicitly refused to complete the questionnaire, resulting in a refusal rate of 0.8%. The main reason for refusal was “tight academic schedule.” All six contacted universities agreed to participate in this study, yielding an institutional response rate of 100%. Ultimately, 1,436 valid questionnaires were collected, with an effective questionnaire recovery rate of 95.73%.

### 2.5 Statistical analysis

Data entry was performed by two individuals, who entered the collected survey data into SPSS 21.0 software for statistical analysis. Non-parametric tests were employed for descriptive statistics. Univariate analysis was first performed using the chi-square test to examine factors influencing college students' rational medication literacy; significant factors were subsequently entered into a binary logistic regression model for multivariate analysis. Structural equation modeling was applied to explore the pathways between these factors and medication literacy. The significance level was set at α = 0.05 for all tests, with all tests being two-tailed.

## 3 Results

### 3.1 Demographic characteristics

A total of 1,500 questionnaires were distributed for this survey, with 1,436 returned, yielding a response rate of 95.73%. Hangzhou Normal University had the largest number of respondents (19.08%), while Zhejiang Chinese Medical University had the smallest (14.35%). Overall, the distribution of respondents was relatively balanced. The survey participants were predominantly female (854, 59.47%) and urban students (834, 58.08%), with a higher proportion of juniors (485, 33.77%) and science and engineering majors (451, 31.41%). The monthly household income of respondents primarily fell within the range of 2,000–7,999 yuan (53.41%), while monthly living expenses were concentrated between 1,000–2,999 yuan (80.64%).

### 3.2 College students' medication literacy scores

The results indicated that the mean medication literacy score of the surveyed college students was 11.32 ± 4.75, with 796 participants (55.43%) achieving high-level literacy (score ≥ 13). Among the four dimensions of medication literacy, the non-prescription drug dimension received the highest mean score (3.65 ± 1.73); the drug advertisement dimension had the lowest (1.63 ± 1.04) (Table 1).

**Table 1:** College students' medication literacy scores (*n* = 1,436).

Dimensions	Minimum value	Maximum value	*M ±SD*
Vocabulary	0	5	3.61 ± 1.53
Non-prescription drugs	0	5	3.65 ± 1.73
Drug advertisements	0	3	1.63 ± 1.04
Prescription drugs	0	4	2.44 ± 1.40
Total score	0	17	11.32 ± 4.75

### 3.3 Levels of rational medication literacy among college students with different characteristics

The results indicated that the Communication University of Zhejiang had the highest proportion of high-level literacy; female students showed a significantly higher rate than males; juniors and seniors exhibited a significantly higher rate than freshmen and sophomores; medical majors displayed a significantly higher rate than non-medical majors; students from cities or county-town areas had a significantly higher rate than those from townships or rural villages; the highest literacy rate appeared in households with a monthly per-capita income of 5,000–7,999 yuan; students whose monthly living expenses were 1,000–2,999 yuan scored significantly higher than those spending below 1,000 or above 3,000 yuan; students whose fathers held bachelor's or associate degrees demonstrated a significantly higher rate than those whose fathers held master's or higher degrees; students whose mothers had completed high school or technical secondary school showed a significantly higher rate than those whose mothers held master's or higher degrees; literacy was significantly higher among participants whose primary family members were not employed in medical or pharmaceutical fields; literacy was higher when primary family members were taking medication; students who had not taken courses on rational medication use exhibited a significantly higher rate; the rate peaked among those who perceived their academic workload as heavy; students with part-time work experience showed a significantly higher rate; and those with high social-support levels scored significantly higher than those with low levels. All differences were statistically significant (*p* < 0.05) (Table 2).

**Table 2:** Comparative analysis of rational medication literacy levels among college students with different characteristics.

Variable groups	*N* (%)	Medication literacy level [*n* (%)]		χ^2^	*p*
		Low level	High level		
University attended					
Zhejiang University	241 (16.78)	136 (56.43)	105 (43.57)	66.071	<0.001
Hangzhou Dianzi University	210 (14.62)	80 (38.10)	130 (61.90)		
Zhejiang Gongshang University	242 (16.85)	113 (46.69)	129 (53.31)		
Hangzhou Normal University	274 (19.08)	159 (58.03)	115 (41.97)		
Zhejiang Chinese Medical University	206 (14.35)	69 (33.50)	137 (66.50)		
Communication University of Zhejiang	263 (18.32)	83 (31.56)	180 (68.44)		
Gender					
Male	582 (40.53)	316 (54.30)	266 (45.70)	37.482	<0.001
Female	854 (59.47)	324 (37.94)	530 (62.06)		
Grade attended					
Freshman	274 (19.08)	135 (49.27)	139 (50.73)	12.511	0.014
Sophomore	257 (17.90)	128 (49.81)	129 (50.19)		
Junior	485 (33.77)	200 (41.24)	285 (58.76)		
Senior	313 (21.80)	123 (39.30)	190 (60.70)		
Fifth	107 (7.45)	54 (50.47)	53 (49.53)		
Major type					
Medical	327 (22.77)	127 (38.84)	200 (61.16)	32.223	<0.001
Literature and history	374 (26.04)	154 (41.18)	220 (58.82)		
Science and engineering	451 (31.41)	194 (43.02)	257 (56.98)		
Arts and physical education	124 (8.64)	81 (65.32)	43 (34.68)		
Others	160 (11.14)	84 (52.50)	76 (47.50)		
Home address					
Township/Rural village	602(41.92)	293 (48.67)	309 (51.33)	7.063	0.008
City/County town	834 (58.08)	347 (41.61)	487 (58.39)		
Monthly per capita household income					
<2,000 yuan	109 (7.59)	74 (67.89)	35 (32.11)	29.617	<0.001
2,000–4,999 yuan	351 (24.44)	152 (43.30)	199 (56.70)		
5,000–7,999 yuan	416 (28.98)	162 (38.94)	254 (61.06)		
8,000–9,999 yuan	293 (20.40)	131 (44.71)	162 (55.29)		
≥10,000 yuan	267 (18.59)	121 (45.32)	146 (54.68)		
Monthly living expenses					
<1,000 yuan	120 (8.36)	93 (77.50)	27 (22.50)	86.193	<0.001
1,000–1,999 yuan	636 (44.29)	244 (38.36)	392 (61.64)		
2,000–2,999 yuan	522 (36.35)	206 (39.46)	316 (60.54)		
≥3,000 yuan	158 (11.00)	97 (61.39)	61 (38.61)		
Father's educational level					
Junior high school and below	573 (39.90)	245 (42.76)	328 (57.24)	35.889	0.001
High school/Vocational school	390 (27.16)	174 (44.62)	216 (55.38)		
College/Undergraduate	388 (27.02)	157 (40.46)	231 (59.54)		
Graduate school and above	85 (5.92)	64 (75.29)	21 (24.71)		
Mother's educational level					
Junior high school and below	632 (44.01)	267 (42.25)	365 (57.75)	56.491	<0.001
High school/Vocational school	394 (27.44)	157 (39.85)	237 (60.15)		
College/Undergraduate	337 (23.47)	153 (45.40)	184 (54.60)		
Graduate school and above	73 (5.08)	63 (86.30)	10 (13.70)		
Employment status of household members					
Yes	240 (16.72)	140 (58.33)	100 (41.67)	66.700	<0.001
No	1,123 (78.20)	442 (39.36)	681 (60.64)		
Not sure	73 (5.08)	58 (79.45)	15 (20.55)		
Long-term medication use among household members					
Yes	657 (45.75)	225 (34.25)	432 (65.75)	69.475	<0.001
No	675 (47.01)	340 (50.37)	335 (49.63)		
Not sure	104 (7.24)	75 (72.12)	29 (27.88)		
Elective course selection status					
Yes	453 (31.55)	226 (49.89)	227 (50.11)	7.585	0.006
No	983 (68.45)	414 (42.12)	569 (57.88)		
Academic workload					
Very demanding	201 (14.00)	120 (59.70)	81 (40.30)	87.967	<0.001
Fairly demanding	631 (43.94)	234 (37.08)	397 (62.92)		
Average	438 (30.50)	173 (39.50)	265 (60.50)		
Fairly easy	125 (8.70)	75 (60.00)	50 (40.00)		
Very easy	41 (2.86)	38 (92.68)	3 (7.32)		
Part-time work experience					
Yes	610 (42.48)	248 (40.66)	362 (59.34)	6.571	0.010
No	826 (57.52)	392 (47.46)	434 (52.54)		
Social support					
Low	80 (5.57)	59 (73.75)	21 (26.25)	32.063	0.001
Medium	1,230 (85.65)	536 (43.58)	694 (56.42)		
High	126 (8.78)	45 (35.71)	81 (64.29)		

### 3.4 Factors influencing college students' medication literacy levels

Using medication literacy (low/high) as a binary dependent variable, where low level was coded as 0 and high level was coded as 1, binary logistic regression analysis was conducted with factors that were statistically significant in univariate analysis as independent variables (Supplementary material). The results indicated that the primary factors influencing college students' rational medication literacy in Zhejiang Province included university attended, gender, major type, monthly living expenses, mother's educational level, employment status of household members, long-term medication use among household members, elective course selection status, academic workload, part-time work experience, and social support. All differences were statistically significant (*p* < 0.05) (Table 3).

**Table 3:** Binary logistic regression analysis of factors influencing college students' medication literacy levels.

Variable groups	β	S.E.	Waldχ^2^	*p*	OR	95% CI	
University attended (reference: Zhejiang University)			27.208	<0.001	
Hangzhou Dianzi University	0.645	0.229	7.957	0.005	1.905	1.217	2.982
Zhejiang Gongshang University	0.191	0.220	0.753	0.385	1.210	0.786	1.863
Hangzhou Normal University	−0.046	0.219	0.044	0.833	0.955	0.622	1.466
Zhejiang Chinese Medical University	0.421	0.236	3.191	0.074	1.524	0.960	2.420
Communication University of Zhejiang	1.006	0.246	16.741	<0.001	2.733	1.689	4.425
Gender (reference: Male)	0.452	0.137	10.859	0.001	1.571	1.201	2.055
Grade attended (reference: Freshman)			5.318	0.256	
Sophomore	−0.072	0.208	0.118	0.731	0.931	0.619	1.400
Junior	0.225	0.188	1.429	0.232	1.252	0.866	1.809
Senior	0.341	0.211	2.598	0.107	1.406	0.929	2.127
Fifth	0.126	0.278	0.206	0.650	1.134	0.658	1.955
Major type (reference: Medical)			37.732	<0.001	
Literature and History	−0.826	0.215	14.705	<0.001	0.438	0.287	0.668
Science and Engineering	−0.398	0.196	4.137	0.042	0.671	0.457	0.986
Arts and Physical Education	−1.757	0.309	32.275	<0.001	0.172	0.094	0.316
Others	−0.939	0.253	13.819	<0.001	0.391	0.238	0.642
Home address (reference: Township/Rural village)	0.167	0.146	1.318	0.251	1.182	0.888	1.574
Monthly per capita household income (reference: <2,000 yuan)			3.857	0.426	
2,000–4,999 yuan	0.051	0.295	0.030	0.862	1.053	0.591	1.875
5,000–7,999 yuan	0.350	0.296	1.397	0.237	1.420	0.794	2.538
8,000–9,999 yuan	0.120	0.313	0.147	0.701	1.128	0.611	2.081
≥10,000 yuan	0.222	0.326	0.465	0.495	1.249	0.659	2.367
Monthly living expenses (reference: <1,000 yuan)			24.950	<0.001	
1,000–1,999 yuan	1.120	0.282	15.790	<0.001	3.065	1.764	5.325
2,000–2,999 yuan	1.080	0.302	12.757	<0.001	2.943	1.628	5.323
≥3,000 yuan	0.404	0.354	1.307	0.253	1.498	0.749	2.997
Father's educational level (reference: Junior high school and below)			5.295	0.151	
High school/Vocational school	−0.145	0.172	0.717	0.397	0.865	0.618	1.211
College/Undergraduate	0.235	0.210	1.255	0.263	1.265	0.838	1.909
Graduate school and above	−0.338	0.365	0.855	0.355	0.713	0.349	1.460
Mother's educational level (reference: Junior high school and below)			10.614	0.014	
High school/Vocational school	0.177	0.170	1.085	0.298	1.193	0.856	1.664
College/Undergraduate	−0.155	0.211	0.544	0.461	0.856	0.566	1.294
Graduate school and above	−1.160	0.439	6.973	0.008	0.313	0.132	0.741
Employment status of household members (reference: Yes)			15.185	0.001	
No	0.436	0.178	5.996	0.014	1.547	1.091	2.193
Not sure	−0.785	0.388	4.091	0.043	0.456	0.213	0.976
Long–term medication use among household members (reference: Yes)			37.155	<0.001	
No	−0.735	0.131	31.726	<0.001	0.479	0.371	0.619
Not sure	−0.987	0.278	12.622	<0.001	0.373	0.216	0.642
Elective course selection status (reference: Yes)	0.335	0.154	4.726	0.030	1.397	1.033	1.890
Academic workload (reference: Very demanding)			22.182	<0.001	
Fairly demanding	0.530	0.196	7.326	0.007	1.698	1.157	2.493
Average	0.308	0.207	2.203	0.138	1.360	0.906	2.042
Fairly easy	−0.185	0.277	0.448	0.504	0.831	0.483	1.430
Very easy	−1.592	0.670	5.653	0.017	0.203	0.055	0.756
Part–time work experience (reference: Yes)	−0.359	0.130	7.614	0.006	0.698	0.541	0.901
Social support (reference: Low)			6.543	0.038	
Medium	0.724	0.308	5.528	0.019	2.063	1.128	3.773
High	0.923	0.371	6.187	0.013	2.517	1.216	5.208
Constant	−2.052	0.465	19.465	<0.001	0.128		

### 3.5 Path analysis of factors influencing college students' medication literacy

**Figure 1:**
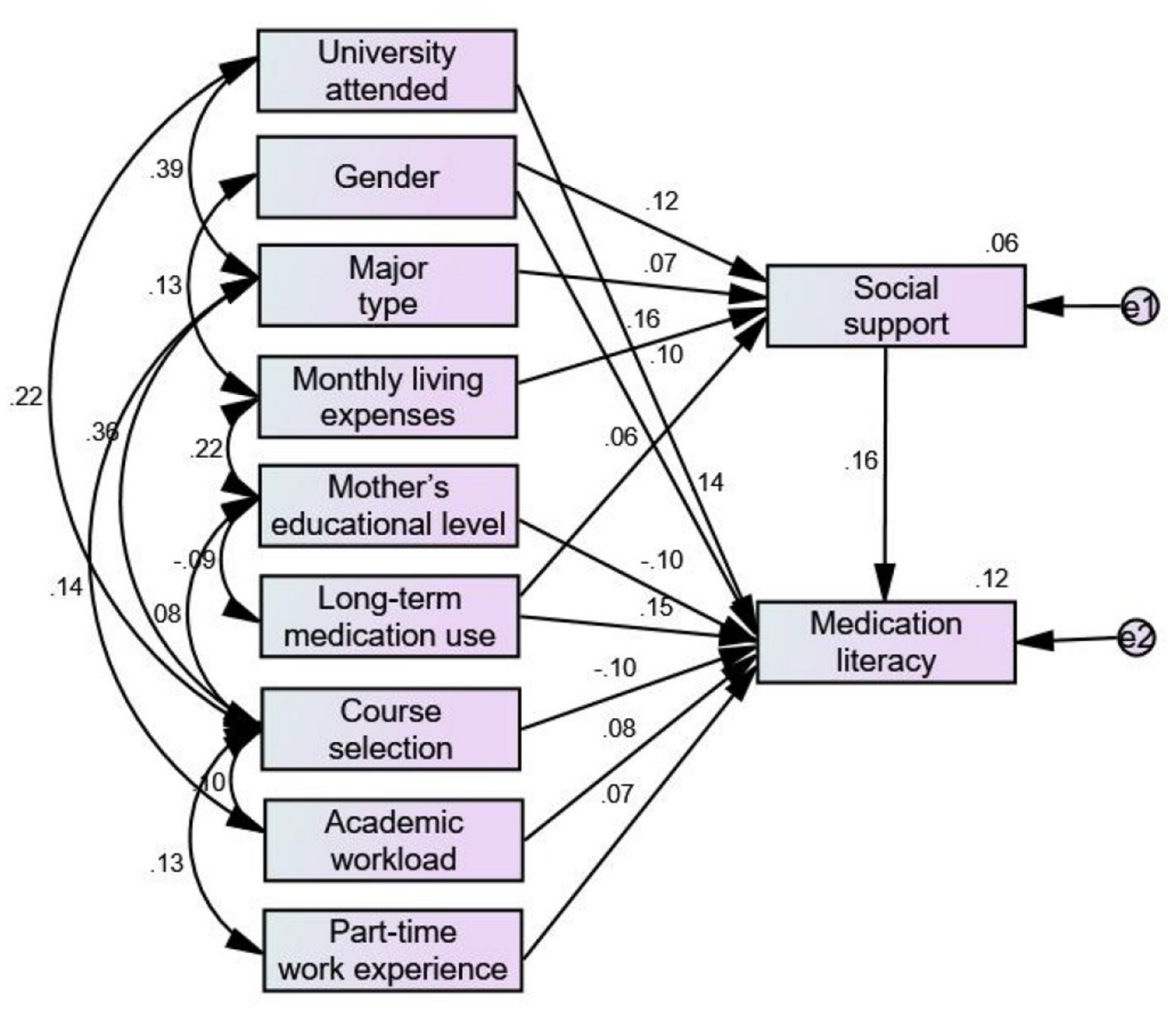
Standardized path coefficients of the revised model.

Based on regression analysis results and relevant professional knowledge, using the level of medication literacy as the dependent variable and social support as the mediating variable, a structural equation model was constructed to identify factors influencing college students' rational medication literacy level (Supplementary material). The model was validated using maximum likelihood estimation. After modifying and removing non-significant paths, the model demonstrated good fit (14–16) (Table 4). The standardized path coefficients of the modified structural equation model for factors influencing college students' rational medication literacy were presented in Figure 1. The effects of each variable on medication literacy were categorized into direct, indirect, and total effects. The total effect sizes of variables on medication literacy were ranked by absolute value from largest to smallest as follows: long-term medication use (0.162), gender (0.160), social support (0.158), mother's educational level (−0.104), university attended (0.100), course selection (−0.095), academic workload (0.081), part-time work experience (0.073), monthly living expenses (0.026), major type (0.012). Among these variables, mother's educational level and course selection exerted negative effects, while the remaining variables exerted positive effects (Table 5).

**Table 4:** Adjusted model fit.

Fitting index	Reference value	Model fit value
χ^2^/df	<3 ideal value; <5 relatively ideal	3.426
RMSEA	<0.05 good fit; <0.08 acceptable fit	0.041
GFI	>0.85 acceptable; >0.90 good	0.986
AGFI	>0.85 acceptable; >0.90 good	0.972
NFI	>0.85 acceptable; >0.90 good	0.891
CFI	>0.85 acceptable; >0.90 good	0.918
IFI	>0.85 acceptable; >0.90 good	0.920
TLI	>0.85 acceptable; >0.90 good	0.864

RMSEA, root mean square error of approximation; GFI, goodness of fit index; AGFI, adjusted goodness of fit index; NFI, normed fit index; CFI, comparative fit index; IFI, incremental fit index; TLI, Tucker-Lewis index; df, degree of freedom.

**Table 5:** Summary of the effects of various variables on medication literacy.

Pathways	Direct effect	Indirect effect	Total effect
University attended → Medication literacy	0.100	–	0.100
Gender → Medication literacy	0.140	0.020	0.160
Major type → Medication literacy	–	0.012	0.112
Monthly living expenses → Medication literacy	–	0.026	0.026
Mother's educational level → Medication literacy	−0.104	–	−0.104
Long-term medication use → Medication literacy	0.153	0.009	0.162
Course selection → Medication literacy	−0.095	–	−0.095
Academic workload → Medication literacy	0.081	–	0.081
Part-time work experience → Medication literacy	0.073	–	0.073
Social support → Medication literacy	0.158	–	0.158

Using the Bootstrap method to examine the mediating effect revealed that social support partially mediated the relationships between gender, major type, monthly living expenses, long-term medication use, and medication literacy. The 95% confidence interval for the mediating effect did not include zero (Table 6).

**Table 6:** Results of the mediation analysis.

Pathways	Effect value	95%CI		p
		Lower	Upper	
Gender → Social support → Medication literacy	0.020	0.011	0.029	0.010
Major type → Social support → Medication literacy	0.012	0.004	0.019	0.010
Monthly living expenses → Social support → Medication literacy	0.026	0.017	0.037	0.010
Long-term medication use → Social support → Medication literacy	0.009	0.002	0.016	0.026

### 3.6 Health education on rational medication use among college students

#### 3.6.1 Implementation status of rational medication use courses

**Figure 2:**
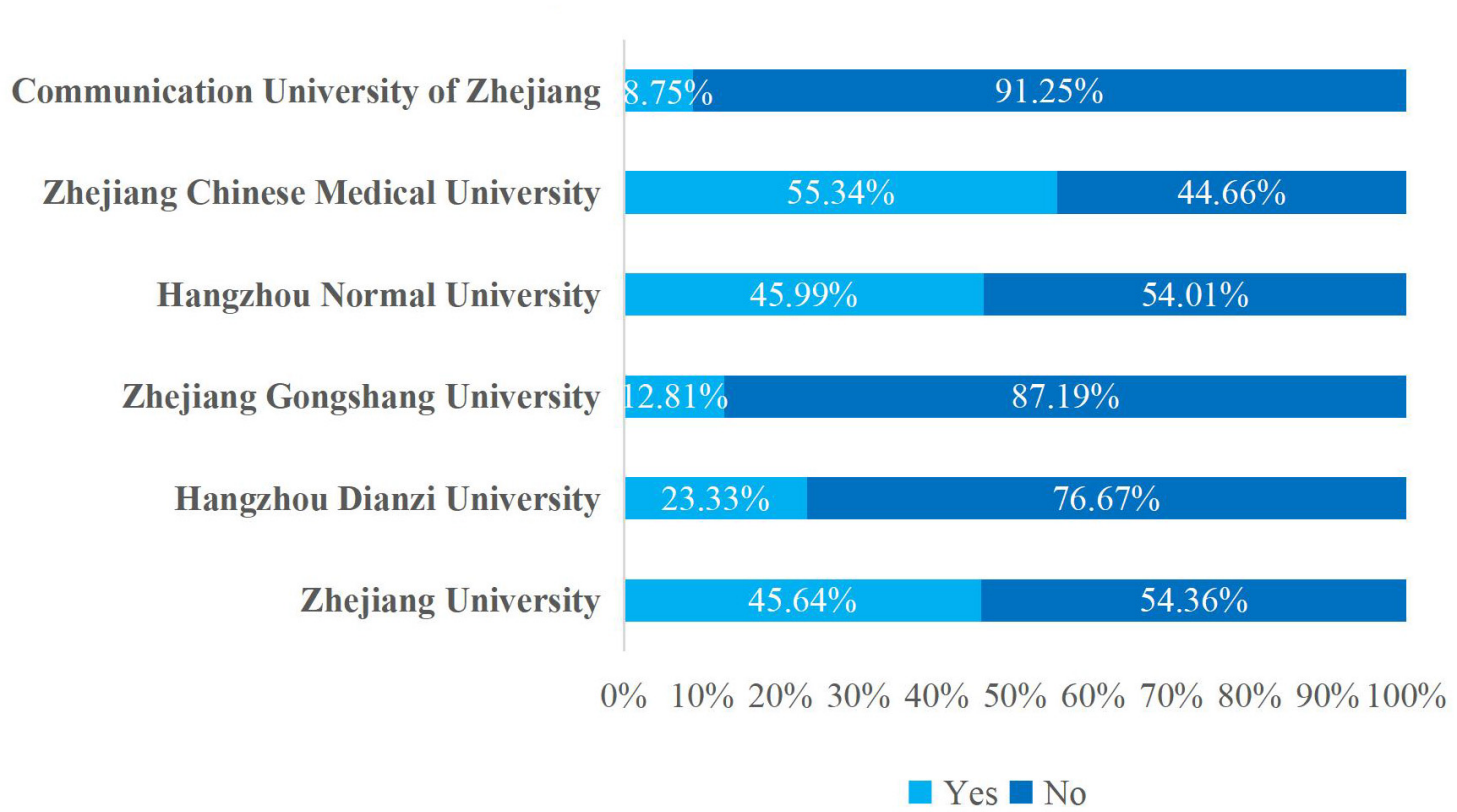
Elective course selection for rational medication use among college students at various universities.

All six surveyed universities offered courses on rational medication use, but overall enrollment rates remained low. Among the surveyed institutions, Zhejiang Chinese Medical University had the highest proportion of students who had taken rational medication use courses, with 114 students (55.34%). Hangzhou Normal University followed with 126 students (45.99%), while Zhejiang University of Media and Communications had the lowest number at 23 students (8.75%). The survey results showed a strong correlation with university type, with medical institutions and comprehensive universities demonstrating significantly higher rates of offering courses related to rational medication use (Figure 2).

#### 3.6.2 Sources for acquiring knowledge on rational medication use

**Figure 3:**
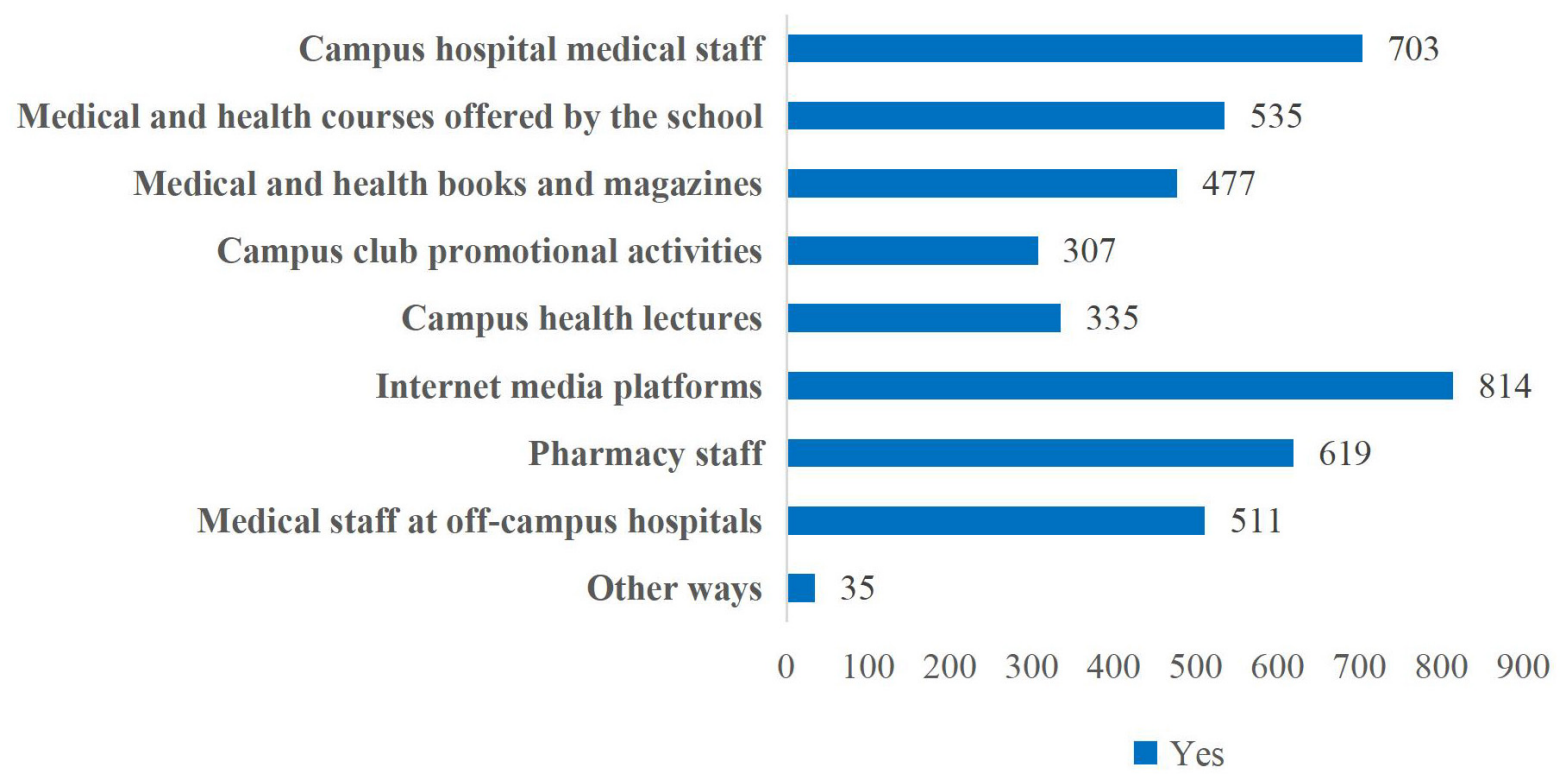
Distribution chart of respondents' primary channels for acquiring knowledge on rational medication use.

Internet media platforms such as WeChat, Douyin, and Xiaohongshu were the primary sources for college students to obtain medication knowledge, with 814 students (56.69%) selecting this option. Second were campus hospital medical staff, selected by 703 students (48.96%). The least popular sources were campus club promotional activities and campus health lectures, each chosen by fewer than one-quarter of respondents—less than 359 students. Notably, a significant number of students also obtained medication knowledge through social channels such as pharmacy staff (619 students, 43.11%) and medical staff at off-campus hospitals (511 students, 35.58%) (Figure 3).

## 4 Discussion

This survey indicated that 796 individuals (55.43%) possessed a high level of medication literacy, suggesting a substantial number of college students in Zhejiang Province demonstrated advanced medication literacy. However, the average medication literacy score was 11.32 points, falling below the 13-point threshold separating low-level from high-level literacy. This finding aligned with the results of studies by Li et al. (7) and Zhang et al. (17). Therefore, there remained considerable room for improvement in the rational medication literacy levels among college students in Zhejiang Province. The drug advertisements received the lowest average score, probably because their exaggerated or misleading claims undermined students' understanding of pharmaceutical knowledge (18).

The results of this study showed that the long-term use of medication by family members had the strongest correlation with students' medication literacy levels, and was positively associated (0.162), meaning that college students who had family members taking medication long-term tended to exhibit higher medication literacy. This is likely related to the fact that this group frequently comes into contact with medication information due to long-term attention to the health of family members. However, some studies indicate that long-term medication use may cause patients or their family members to become too familiar with commonly used drugs and thus tend to ignore reading instructions, paying attention to contraindications and adverse reactions, which may, to some extent, weaken their awareness of prudent medication use (19). Gender was not only directly related to medication literacy (0.160), but also indirectly affected it through social support as the mediator (0.020). In this study, female students generally exhibited higher medication literacy levels than male students, which is likely related to women typically paying more attention to details and health issues (such as adverse drug reactions) (20). Moreover, females tended to be better at seeking and utilizing emotional and informational support from others, which helped them better understand and manage medication information. Social support was positively associated with medication literacy (0.158), which is consistent with previous research findings (21). The social etiology perspective suggests that social support can generally enhance people's confidence, sense of stability, self-efficacy, and sense of control over their environment (22). Therefore, college students who received higher social support tended to show greater initiative and held a more positive attitude toward various learning content, including medication knowledge. Mother's level of education was negatively associated with students' medication literacy (−0.101), which differs from the findings of Zheng et al. (23) on health literacy. This discrepancy is likely because medication literacy relies more on daily practical supervision rather than merely the transmission of knowledge. At the same time, highly educated mothers often have more demanding career responsibilities, which likely reduces the time they can directly supervise and guide their children's daily medication behaviors. Of course, this complex relationship still requires further research to explore. There was a positive correlation (0.100) between the university attended and the level of medication literacy, which is likely related to the emphasis on different disciplines in various universities. Students at comprehensive or specialized universities that focus on medical education generally have more opportunities and an environment to access pharmaceutical knowledge. For example, as a medical university, Zhejiang Chinese Medical University students showed a relatively high level of medication literacy in this survey. Course selection was negatively associated with medication literacy (−0.095), meaning that students who had not taken rational medication courses actually exhibited higher medication literacy, which is inconsistent with the findings of Supapaan et al. (24). One possible explanation is that most universities offer rational medication courses as general elective courses, focus on short-term conveyance of theoretical knowledge in a one-way manner, and lack corresponding practical sessions. This results in students having limited ability to apply knowledge, and the generally low importance placed on elective courses makes it difficult for the knowledge learned to be internalized, preventing the formation of long-term stable literacy. Academic workload was positively associated with the level of medication literacy (0.081), meaning that students who perceived their academic workload as heavy exhibited higher medication literacy than those who perceived it as light. This contrasts with the findings of Liu et al. (25), who found that when students perceived their academic workload as light, they tended to demonstrate stronger coping skills and self-planning abilities, thereby possessing higher health literacy. Part-time work experience was positively associated with the level of medication literacy (0.073). Previous studies have shown that college students' motivations for choosing part-time jobs are mainly due to economic pressure and employment pressure (26), which may suggest that students with part-time work experience tend to plan their future more proactively, possess stronger autonomy and execution ability, and are therefore more likely to proactively explore knowledge areas related to medication literacy. Monthly living expenses played an indirect role in medication literacy through social support (0.026). Students with monthly living expenses in the 1,000–2,999 yuan range usually have relatively affluent economic conditions and stable family support, are more active in social activities, and thus have higher levels of social support, allowing them to obtain more medication information through peer communication and guidance from relatives and friends, thereby indirectly improving their medication literacy. The type of major also indirectly affected medication literacy through social support (0.012). Medical students have more opportunities to be exposed to pharmaceutical knowledge in their daily coursework (27), so their medication literacy is significantly higher than that of students in other majors. At the same time, the systematic pharmaceutical knowledge and practical training provided by medical education give medical students an advantage over students in other majors in terms of drug cognition and awareness of rational drug use.

This survey found that college students primarily obtained medication knowledge through internet media platforms. Consequently, students could fully leverage existing online resources for self-directed learning. They could follow science-based content published by the National Medical Products Administration (NMPA), the World Health Organization (WHO), or university medical-school websites to access authoritative and accurate medication information, and they could also study pharmacy-related courses on campus learning platforms such as MOOCs. To benefit most from these resources, college students should cultivate critical-thinking skills, learn to identify exaggerated claims in pharmaceutical advertisements, master techniques for retrieving medication information, and avoid common medication misconceptions.

This survey also found that the implementation of rational-medication courses varied across universities and that these courses had not achieved the expected educational outcomes. It is therefore recommended that general-education courses on rational medication adopt a model that combines formal classroom instruction with extracurricular activities. The programme should focus on areas of student interest—such as club activities, academic competitions, and social practice—to blend theory with application. Specific initiatives could include: ① utilizing health-related clubs to organize medication-health activities; ② incorporating health-science popularization into academic competitions; ③ collaborating with medical schools to run “Safe Medication Use” workshops; ④ organizing student visits to pharmaceutical companies or hospital pharmacies. The study found that 48.96% of students primarily obtained their medication knowledge from the medical staff in the campus clinic and that there was a close relationship between the construction of the campus clinic and the level of medication literacy (28). It is recommended that the campus clinic, while providing healthcare services to teachers and students, should also strengthen its functional role in higher education by spreading health knowledge and cultivating and guiding the health awareness of teachers and students.

This study found differences among various types of universities in the offering of rational medication use courses and students' course selection. Therefore, it is recommended that qualified universities, especially medical colleges, rely on this provincial shared platform to include high-quality courses and lecture resources related to rational drug use in the inter-university shared list; non-medical universities can proactively connect with medical universities to carry out teaching cooperation, using methods such as online elective courses, offline lectures, and joint practical training to make up for their own lack of professional resources, thereby achieving inter-university resource complementarity and jointly creating a good environment for cultivating rational drug use literacy on campus. This study also found that social channels—such as internet media platforms, pharmacy staff, and healthcare personnel at off-campus hospitals—served as important avenues for college students to acquire medication knowledge. It is therefore imperative to mobilize social forces to participate in campus-based medication-health-promotion initiatives, integrating both online and offline channels. For instance, universities can collaborate online with influential promoters of medication knowledge on platforms such as WeChat Official Accounts and Douyin; these influencers would regularly deliver evidence-based information about safe medication use to students and spark their enthusiasm for learning (29). Offline, partnerships with nearby chain pharmacies, pharmaceutical companies, and non-profit organizations can be leveraged to organize “Safe Medication Awareness Weeks” that provide educational materials (handbooks, pill organizers, etc.) and free consultation services.

## 5 Limitations

A limitation of this study is that the selected universities did not include private institutions or junior colleges, potentially introducing certain limitations to the findings. To comprehensively assess medication literacy among college students and further validate the conclusions, future research may consider expanding the scope of institutions surveyed. Furthermore, this survey constituted a cross-sectional study: data were collected at a single point in time, so its explanatory power regarding underlying causes, motivations, or processes was limited. It could not establish causal relationships between the influencing factors and college students' medication literacy. Longitudinal research is therefore required to investigate these relationships in depth. The closed-ended questions in the questionnaire are difficult to capture the complex attitudes and underlying behavioral motivations of respondents; self-reported data are prone to response biases such as social desirability bias, and the way questions are phrased and ordered may also unintentionally influence responses. Future research could improve this study by combining in-depth interviews and longitudinal tracking methods.

## 6 Conclusion

The level of rational medication literacy among college students in Zhejiang Province requires improvement, and the development of rational medication courses and teaching effectiveness in higher education institutions also need further enhancement. It is recommended to establish a health education network for rational medication in universities by focusing on three aspects: government guidance, institutional promotion, and self-improvement. This initiative aims to institutionalize the cultivation of awareness regarding safe and rational medication use, thereby enhancing students' health literacy, particularly in the area of rational medication practices.

## Data Availability

The original contributions presented in the study are included in the article/Supplementary material, further inquiries can be directed to the corresponding author.
