# Supplementary material for: Research on influencing factors and improvement paths of rational medication literacy among college students: a cross-sectional survey in Zhejiang province
Source: Front Public Health. 2026 Apr 10;14:1775243. doi: 10.3389/fpubh.2026.1775243 (PMC13106574; doi:10.3389/fpubh.2026.1775243)
Supplement: Supplementary file 1 [file Data_Sheet_1.pdf]

Appendix 1

Variable assignment for binary logistic regression analysis.

Factors	Variable name	Assignment description
University attended	X1	“Zhejiang University” = 0
		“Hangzhou Dianzi University” = (1, 0, 0, 0, 0)
		“Zhejiang Gongshang University” = (0, 1, 0, 0, 0)
		“Hangzhou Normal University” = (0, 0, 1, 0, 0)
		“Zhejiang Chinese Medical University” = (0, 0, 0, 1, 0)
		“Communication University of Zhejiang” = (0, 0, 0, 0, 1)
Gender	X2	“Male” = 0
		“Female” = 1
Grade attended	X3	“Freshman” = 0
		“Sophomore” = (1, 0, 0, 0)
		“Junior” = (0, 1, 0, 0)
		“Senior” = (0, 0, 1, 0)
		“Fifth” = (0, 0, 0, 1)
Major type	X4	“Medical” = 0
		“Literature and History” = (1, 0, 0, 0)
		“Science and Engineering” = (0, 1, 0, 0)
		“Arts and Physical Education” = (0, 0, 1, 0)
		“Others” = (0, 0, 0, 1)
Home address	X5	“Township/Rural village” = 0
		“City/County town” = 1
Monthly per capita household income	X6	“<2000 yuan” = 0
		“2000-4999 yuan” = (1, 0, 0, 0)
		“5000-7999 yuan” = (0, 1, 0, 0)
		“8000-9999 yuan” = (0, 0, 1, 0)
		“≥10000 yuan” = (0, 0, 0, 1)
Monthly living expenses	X7	“<1000 yuan” = 0
		“1000-1999 yuan” = (1, 0, 0)
		“2000-2999 yuan” = (0, 1, 0)
		“≥3000 yuan” = (0, 0, 1)
Father’s educational level	X8	“Junior high school and below” = 0
		“High school/Vocational school” = (1, 0, 0)
		“College/Undergraduate” = (0, 1, 0)
		“Graduate school and above” = (0, 0, 1)
Mother’s educational level	X9	“Junior high school and below” = 0
		“High school/Vocational school” = (1, 0, 0)
		“College/Undergraduate” = (0, 1, 0)
		“Graduate school and above” = (0, 0, 1)
Employment status of household members	X10	“Yes” = 0
		“No” = (1, 0)
		“Not sure” = (0, 1)

Long-term medication use among household members	X11	"Yes" = 0
		"No" = (1, 0)
		"Not sure" = (0, 1)
Elective course selection status	X12	"Yes" = 0
		"No" = 1
Academic workload	X13	"Very demanding" = 0
		"Fairly demanding" = (1, 0, 0, 0)
		"Average" = (0, 1, 0, 0)
		"Fairly easy" = (0, 0, 1, 0)
		"Very easy" = (0, 0, 0, 1)
Part-time work experience	X14	"Yes" = 0
		"No" = 1
Social support	X15	"Low" = 0
		"Medium" = (1, 0)
		"High" = (0, 1)
Medication literacy	X16	"Low level" = 0
		"High level" = 1

Appendix 2

Variable assignment for structural equation modeling.

Factors	Variable name	Assignment description
University attended	X1	"Non-medical universities" = 1
		"Medical universities" = 2
Gender	X2	"Male" = 1
		"Female" = 2
Grade attended	X3	"Freshman" = 1
		"Sophomore" = 2
		"Junior" = 3
		"Senior" = 4
		"Fifth" = 5
Major type	X4	"Non-medical major" = 1
		"Medical major" = 2
Home address	X5	"Township/Rural village" = 1
		"City/County town" = 2
Monthly per capita household income	X6	"<2000 yuan" = 1
		"2000-4999 yuan" = 2
		"5000-7999 yuan" = 3
		"8000-9999 yuan" = 4
		"≥10000 yuan" = 5
Monthly living expenses	X7	"<1000 yuan" = 1
		"1000-1999 yuan" = 2
		"2000-2999 yuan" = 3
		"≥3000 yuan" = 4

Father's educational level	X8	"Junior high school and below" = 1
		"High school/Vocational school" = 2
		"College/Undergraduate" = 3
		"Graduate school and above" = 4
Mother's educational level	X9	"Junior high school and below" = 1
		"High school/Vocational school" = 2
		"College/Undergraduate" = 3
		"Graduate school and above" = 4
Employment status of household members	X10	"No" = 1
		"Yes" = 2
Long-term medication use among household members	X11	"No" = 1
		"Yes" = 2
Elective course selection status	X12	"No" = 1
		"Yes" = 2
Academic workload	X13	"Very easy" = 1
		"Fairly easy" = 2
		"Average" = 3
		"Fairly demanding" = 4
		"Very demanding" = 5
Part-time work experience	X14	"No" = 1
		"Yes" = 2
Social support	X15	-
Medication literacy	X16	-
